# Bee-Keeping

**Published:** 1892-07

**Authors:** 


### BEE-KEEPING.

Last year throughout the North and West there was very little profit to be derived from bees, and the two previous years there were but small yields. In conseqence of this, bee-keepers had the "blues," and began to think that bee-keeping was a delusion and a snare—too uncertain to depend upon for a living. Farmers and horticulturists meet with poor seasons, but what would become of this country if they all in a body threw up their occupation ? Manufacturers have to meet disappointments in the sales of their products, and it has to be met in all walks of life.

From reports received it appears that the clouds are lifting, and that the prospect for honey the coming year is brighter than for a year or so past. In some localities white clover grew finely last summer but too late to yield honey, but under favorable conditions may yield this season. Sweet clover is pretty generally introduced throughout the West, and like the children of Israel in Egypt, the more it is persecuted the more it multiplies and grows. It thrives in this sandy soil. A lady pointed to some in her garden saying: "I raise that for my chickens." I have noticed fowls feeding upon it, as it starts earlier than other clovers.

Not long since I met a man who said: "I've been keeping bees for more than forty years but I never could get them to increase to more than four or five colonies until I got the patent hives which have movable frames." Now let us look into the matter and see if we can divine the reason. There is no way by which we can see into the interior of a box hive. We have to depend in a great measure upon guess work. A colony may throw off swarm after swarm until it is literally ruined, and there is no way of preventing it. If it was in a movable frame hive the combs could be lifted out and the queen cells cut off and the second swarm returned, when there would be two big strong colonies able to take care of themselves and yield fair returns to their owner, in lieu of four or five hives in use, paying no rent, and the tenants barely able to live, in most instances dying the following winter.

The moth is a terrible fellow to the box hive man. When a colony loses its queen these gentry come in and find a house well provisioned and furnished. When the owner sees there are no bees coming and going to sting him, he bravely turns up the hive and exclaims: "The plaguey moths have destroyed this colony!" All this time they were as innocent as a man in the moon. It is true that they destroyed the contents of the hive, eating the comb and bee-bread, but if there had been plenty of bees there would not have been room enough for them, for bees do not believe that there is a house large enough for two families —a family of bees and one of moths. If a colony in a movable frame hive loses its queen, the fact can be readily ascertained and another given to them. If a colony needs food it can be supplied. If very weak it can be strengthened with mature brood, or if diseased can be taken care of before the contagion spreads.—*Exchange.*

